# Supplementary figures and images for: A Preclinical Embryonic Zebrafish Xenograft Model to Investigate CAR T Cells in Vivo
Source: Cancers (Basel). 2020 Feb 29;12(3):567. doi: 10.3390/cancers12030567 (PMC7139560; doi:10.3390/cancers12030567)

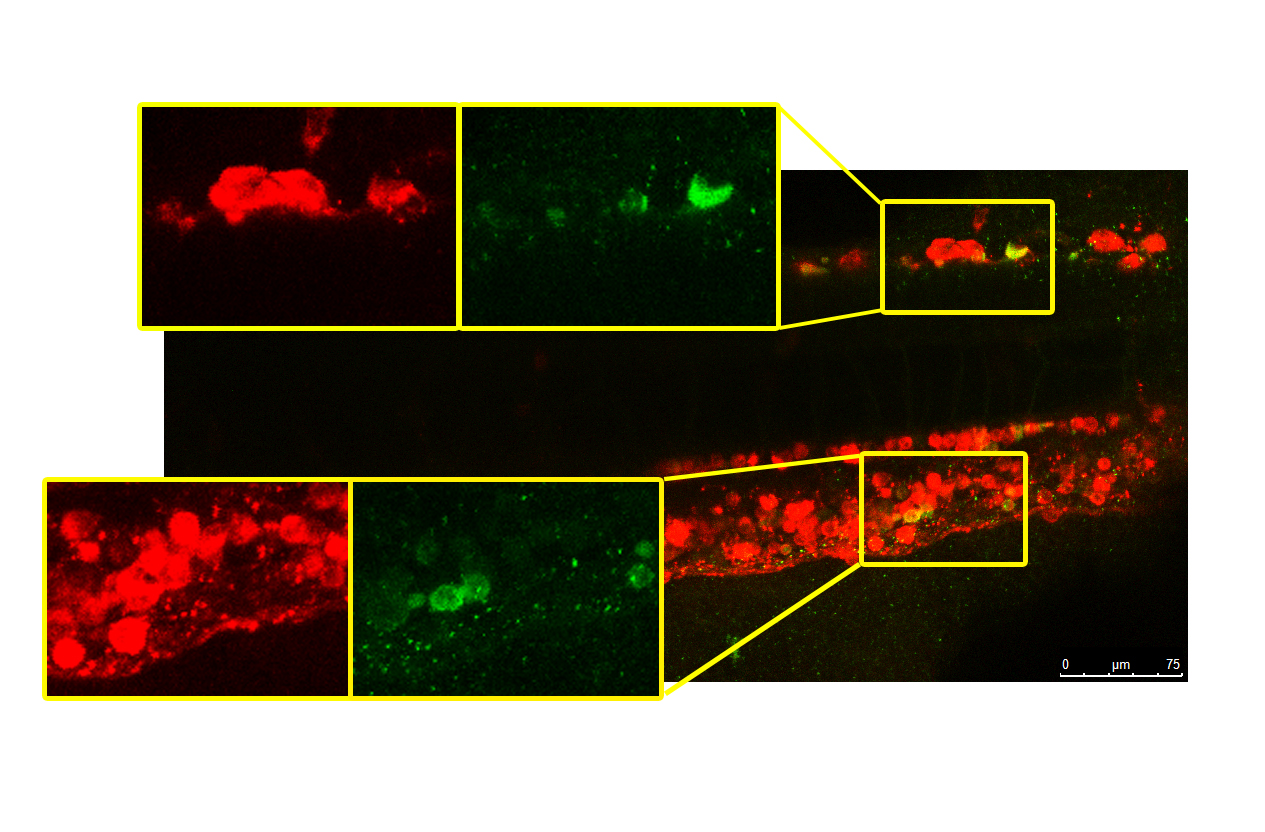

Supplement: Supplementary file 1 [file cancers-12-00567-s001.zip › supplementary files/FigS1.jpg]
